# Highlighting hybridization: a case report of virtual reality-augmented interventions to improve chronic post-stroke recovery

**DOI:** 10.1097/MD.0000000000029357

**Published:** 2022-06-24

**Authors:** Rachel Blanchette Bailey

**Affiliations:** XRHealth, Brookline, MA.

**Keywords:** case report, stroke rehabilitation, telerehabilitation, virtual reality

## Abstract

**Rationale:**

Cerebrovascular accident, or stroke, is a leading cause of serious long-term disability, and incidence is expected to continue rising in the coming years. Stroke-related disability can manifest as motor, sensory, or cognitive impairment, and although current therapies can improve these impairments, many stroke patients are still left with reduced abilities and struggle to maintain independence in their daily lives. Virtual reality (VR) has been proposed as a novel therapeutic tool to treat stroke-related disability, particularly in conjunction with traditional post-stroke therapies.

**Patient concerns:**

Here, we report 1 case of a 75-year-old male with ongoing right upper extremity impairment.

**Diagnosis:**

The patient was previously diagnosed with an ischemic stroke.

**Interventions:**

Seven years post-stroke, during which he did not receive any therapies, and on the recommendation of his neurologist, the patient sought VR therapy as an innovative treatment strategy. His clinicians employed a combination of traditional exercise and VR therapy to improve his strength and mobility.

**Outcomes:**

The combination of traditional and VR therapy was able to provide meaningful improvement to his daily quality of life, even years after the stroke.

**Conclusions:**

These results suggest that combination therapy with VR is a viable tool for treating post-stroke impairment, even several years on from the cerebrovascular accident.

## 1 Introduction

A cerebrovascular accident (CVA), or stroke, is a sudden onset of neurological deficits caused by vascular injury to the brain.^[1]^ Nearly 795,000 people annually in the United States have a stroke, of which 610,000 are the first stroke for the individual.^[2]^ Furthermore, epidemiological predictions forecast that the number of strokes in the United States will continue to rise.^[3]^ A CVA is classified by vascular damage, which can be either ischemic or hemorrhagic. A cerebral blood vessel ruptures in a hemorrhagic stroke, while ischemic stroke (accounting for 87% of strokes^[2]^) results from cerebral vessel blockage.^[1]^ Strokes are the leading cause of serious long-term disability, and stroke-related costs from 2014 to 2015 were approximately $46 billion.^[2]^ The effects of a stroke can include sensory, motor, and cognitive impairment, all of which can impact the ability to perform typical daily activities.^[4]^ The most common manifestation of CVA is hemiparesis or hemiplegia, which is unilateral weakness and movement dysfunction. This motor impairment can range from mild to complete paralysis, and many patients do not regain full movement or function in their upper extremity.^[1]^ There are many interventional strategies to improve upper extremity function post-stroke, which include constraint-induced movement therapy (CIMT), mirror therapy, electrical stimulation, and various exercise approaches.

Technological advances have introduced virtual reality (VR) as a novel therapeutic tool. VR creates a three-dimensional (3D) environment for individuals to experience a modified version of reality. The visual, auditory, and kinesthetic multisensory nature of VR allows patients to be fully immersed in the experience, which can provide a distraction, or allow patients to experience phenomenon that can be therapeutic.^[5,6]^ VR has been successfully utilized for pain management, and as a useful distraction while undergoing certain medical treatments that can be painful or anxiety-inducing.^[6–10]^ Though still in early stages of research, VR as a therapy tool for stroke patients, either as a stand-alone therapy, or in conjunction with more traditional post-stroke therapies, is under investigation.^[11,12]^

A novel software has been developed that guides patients through exercises according to an individualized treatment plan from their medical practitioner. In conjunction with a VR headset, this software has marketing clearance by the US, European, and Israeli regulatory bodies, and enables patients to receive treatment without leaving their home. Training can be initiated remotely by the occupational therapist, using the external control system to load different activities into the digital environment on the patient's headset. The occupational therapist can mirror the patient's headset, while also being able to observe the patient's movements via telehealth. Therapists can give and receive real-time feedback with patients, making adjustments as needed to ensure patients experience the maximum therapeutic benefit. This allows patients to increase physical activity and functionality, thus supporting a more positive quality of life.

Here we describe the case of an individual who sustained a stroke and employed VR-assisted interventions during occupational therapy after more than 7 years without therapy, from which significant improvements were observed.

## 2 Case description

The following is an exploration of 1 episode of treatment for a 75-year-old male who was diagnosed with a cerebrovascular event in 2014. He experienced an ischemic stroke, likely the result of a blood clot related to a diagnosis of hypertension. He received recombinant tissue plasminogen activator at the hospital, and eventually transitioned to an inpatient rehabilitation facility, where he received occupational (OT), physical, and speech language pathology therapies. He returned home without home health services and reported the ability to walk normally, but struggled with aphasia and right upper extremity (RUE) motor impairment.

For 7 years following the stroke, the patient did not obtain any additional therapies, but continued to have annual visits to his neurologist. He did not do any exercise for his RUE impairment, and had mostly adapted to performing daily tasks left-handed, including brushing his teeth and writing his signature. His stroke caused abnormal muscle tone leading to muscle weakness, synergistic movement patterns, and decreased active range of motion with his RUE. Joint contractures were present at his right hand and wrist, but he did have active movement in all 5 digits and a functional grasp between his first and second digits. At the encouragement of his neurologist, he sought VR therapy as an innovative treatment strategy for his ongoing RUE impairment. The goal of using VR-augmented therapy was to improve strength and functional use of his right arm, to better perform life tasks. All patient visits were conducted via telehealth, and he appeared comfortable in his own home as he described post-stroke experiences. The occupational therapist was able to assess his RUE active range of motion (AROM) and muscle tone via observation, and was able to visualize the set-up of his home. This ensured the therapist could make personalized adjustments to the therapy, both preemptively and throughout the course of treatment.

The occupational therapist assisted the patient with developing a comprehensive exercise program for management of RUE muscle tone, as well as improving his strength and AROM, which included understanding the VR applications selected by the occupational therapist and preloaded onto the headset. To begin training, the occupational therapist used the external control system to initiate different applications onto the patient's headset including: “Rotate”, in which the patient used his gaze to track the movements of a flying dragon, which promotes cervical stretching and AROM; “Balloon Blast”, which measures shoulder AROM and aims to improve AROM by playing a game to pop colored balloons with swiping motions; and “Color Match” for upper extremity movements mimicking a boxing motion to strike matching-colored circles and to incorporate a cognitive component to the activities in the digital environment. Following each VR session, the patient could visualize performance and observe changes over time. The occupational therapist selected applications and set parameters, such as grid size and speed, and the patient was expected to dedicate a set amount of time to using his headset each day. In ColorMatch, average response time and average action time were tracked from when the circle lights up, to when the patient initiates movement and the time it takes from when a colored circle lights up, to when the patient touches the circle, respectively.

Over the course of the OT treatment, this patient participated in traditional telehealth interventions, in addition to VR assisted therapy. He underwent 12 treatment sessions over a 2-month period, at a frequency of 2 times per week for 1 month, then once per week for 1 month. In addition to daily use of his VR headset, the patient performed exercises 5 days per week, including weight-bearing through his right arm in the quadruped position, and active, assisted ROM movements of his right arm. By the end of treatment, the patient performed resisted right arm and hand exercises with a 2lb weight and stress ball. The patient also participated in retraining for traditional activities of daily living (ADLs), which included identifying difficult self-care tasks. The occupational therapist provided education for adaptation as well as specific training for the VR therapy body mechanics and techniques. During sessions, the patient could give and receive feedback in real-time, and the cooperative effort allowed the patient to maximize the effect of the VR software. The patient had an average compliance of 90% with his virtual treatment plan, translating to almost daily usage. Not only did this virtual treatment plan allow the patient to independently access his VR headset, the plan was also continuously upgraded throughout his therapy, in order to keep the games in the digital environment challenging and to promote progress.

The patient demonstrated improvement in several areas, and progress was routinely measured with outcome assessments. The Stroke Impact Scale was used to measure the patient's perception of his progress. This self-reporting questionnaire has 59 items categorized into domains (strength, memory, emotion, communication, ADLs/instrumental ADLs (IADLs), mobility, hand function, social participation, and physical). Although the patient did not meet the criteria for a minimally clinically important difference, he did show areas of perceived improvement as evidenced by an increase in Stroke Impact Scale score from 12 to 15. The patient also experienced improved ease with ADL (score improved from 45 to 48) and IADL tasks as he experienced improvements in RUE strength and AROM. Most significantly, at care initiation, the patient rated himself as 75% recovered from the stroke while at discharge, this number had improved to 90%.

Performance based measures and data collected via the VR headset indicated measured progress in the areas of RUE strength, AROM, average response time, and quality of movement. The Hand-to-Head Test was used to measure bilateral upper extremity strength and showed an improvement by 7 additional repetitions with RUE and 3 additional repetitions with left upper extremity (Table 1).

**Table 1:** Performance-based improvements.

Assessment	Baseline	Discharge
Hand-to-head test		
RUE	18	25
LUE	25	28
Color Match (seconds)		
RUE average response time	0.98	0.60
RUE average action time	1.56	0.94
Balloon Blast		
RUE flexion (degrees)	162	172
RUE quality of movement score (%)	84.7	93.2

LUE = left upper extremity, RUE = right upper extremity.

Progress according to the VR headset was monitored weekly, and the patient demonstrated improvements in all measured areas. The speed at which the patient was able to move his right arm in response to a stimulus was measured via the “ColorMatch” application, as average response time and average action time. For the RUE, the average response time decreased from 0.98 seconds to 0.60 seconds, and the average action time of 1.56 seconds decreased to 0.94 seconds by the end of the therapy (Table 1). These data suggest an improved ability to quickly respond to a stimulus when reaching with his right arm. In “Balloon Blast”, the patient demonstrated a 10-degree improvement in shoulder flexion over the course of treatment, improving from 162 to 172 degrees. “Balloon Blast” also measures quality of movement, which evaluates acceleration-deceleration while moving. The first time this patient played Balloon Blast, he had a quality of movement score of 84.7%, that improved to 93.2% by the end of treatment.

## 3 Discussion

This case report demonstrates improved outcome measures, both self-reported, and performance-based, as well as by the VR headset measurements. In addition to this measured progress, the patient was able to note functional ADL and IADL improvements. He had not maintained any program for RUE muscle tone or exercise since his discharge from an inpatient rehabilitation hospital 7 years prior to the current therapy regimen. Previous reports have indicated that VR was not more beneficial than conventional therapy approaches when it comes to upper limb dysfunction.^[4]^ Here, we present a VR-augmented therapy plan, where the patient was treated with a combination of traditional telehealth and VR-therapy, 7 years beyond the original incident. This patient demonstrated measured progress by the end of the treatment plan in RUE strength and AROM, the ability to perform ADL and IADL tasks, and also improved perception of post-stroke recovery. This case provides evidence that VR-augmentation may provide the means of enhancing traditional therapy with new VR techniques, to provide not only measurable, but perceived improvements to patient quality of life, even many years after the initiating event.

Published literature shows that VR therapies provide gains in mobility, functional independence, and quality of life, and furthermore, these gains are not limited to upper extremities.^[13–15]^ While a meta-analysis has indicated that VR rehabilitation provides only moderate improvement over traditional therapies,^[14]^ this case reports provides evidence to support combination, or hybridized interventions that combine conventional treatments with VR-augmented therapies, resulting in improved patient outcomes. Current research is examining the utility of VR not only as therapy but also to identify specific patient populations that may benefit from VR, how to best implement the therapies, and to measure and monitor patients’ response to interventions.^[15–17]^

Another clear benefit to VR-augmented therapy is that, unlike some traditional stroke interventions, which are best applied as soon after the cerebral event as possible,^[18]^ this therapy regiment was implemented 7 years post-stroke, and with essentially no therapies in the intervening years. Despite this, significant mobility gains and quality of life improvements were observed, demonstrating that VR-augmented interventions can potentially benefit many stroke victims, regardless of elapsed time since the stroke. This can not only significantly reduce costs related to strokes, but also provide tangible improvements for the many patients that experience long-term disability due to stroke.^[2]^

Limitations of the method included the inability to manually assess the patient's RUE strength and muscle tone, assessments that are performed routinely at in-person practice settings. Additionally, there is a learning curve at the start of treatment, as initial sessions focus on training and educating patients and caregivers on how to access and utilize the VR headset. This can become increasingly challenging in the case of patients who are not comfortable with new technology.

Throughout the course of care, the patient's progress was monitored using self-reporting questionnaires, performance-based measures, and via real-time monitoring of data from the VR headset. Performance parameters were adjusted to provide a graded, progressive stimulus. For this patient, traditional OT telehealth treatment combined with VR-assisted interventions was successful in treating RUE impairment resulting from a stroke. The addition of VR therapy to the treatment regimen eliminated frustration with tasks that were previously challenging and improved quality of life. Taken together, while more research is required to assess the use of VR-assisted interventions, these data suggest that VR as a treatment modality in combination with conventional therapy is a promising treatment strategy for the post-stroke population.

## 4 Patient perspective

As a result of my stroke 7 years ago, my right arm and hand were significantly restricted. Initial physical therapy and OT right after the stroke helped some, but there were still some activities that I wanted to be easier to do: put on work gloves, do yard work, and shift my sports car while driving. These were my goals for my VR program. The VR exercises improved my ability to manipulate my right hand into a glove. It also helped me to be able to control and direct my electric lawn mower, making it much easier to mow my lawn. As for my sports car, since shifting required dexterity of my right arm and hand, I was afraid I would have to sell my car. But the VR exercises improved the use of my right arm and hand to be able to shift easily, and I can continue to enjoy driving my sports car.

## Acknowledgments

We would like to thank the patient for his informed consent, and commitment to the VR therapy program, and his wife for her support throughout the treatment. JetPub Scientific Communications, LLC supported by funding from XRHealth, provided drafts and editorial assistance to the author during preparation of this manuscript.

## Author contributions

**Formal analysis:** Rachel Bailey.

**Investigation:** Rachel Bailey.

**Writing – original draft:** Rachel Bailey.
